# Roles of Shiga Toxins in Immunopathology

**DOI:** 10.3390/toxins11040212

**Published:** 2019-04-09

**Authors:** Moo-Seung Lee, Vernon L. Tesh

**Affiliations:** 1 Environmental Diseases Research Center, Korea Research Institute of Bioscience and Biotechnology, 125 Gwahak-ro, Daejeon 34141, Korea; 2 Department of Biomolecular Science, KRIBB School of Bioscience, Korea University of Science and Technology (UST), 127 Gajeong-ro, Yuseong-gu, Daejeon 34113, Korea; 3 Department of Microbial Pathogenesis and Immunology, Texas A&M University Health Science Center, Bryan, TX 77807, USA

**Keywords:** Shiga toxins, Shiga toxin types 1 and 2, Shiga toxin-producing *Escherichia coli*, hemolytic uremic syndrome, bacterial toxins, immunopathology

## Abstract

*Shigella* species and Shiga toxin-producing *Escherichia coli* (STEC) are agents of bloody diarrhea that may progress to potentially lethal complications such as diarrhea-associated hemolytic uremic syndrome (D+HUS) and neurological disorders. The bacteria share the ability to produce virulence factors called Shiga toxins (Stxs). Research over the past two decades has identified Stxs as multifunctional toxins capable of inducing cell stress responses in addition to their canonical ribotoxic function inhibiting protein synthesis. Notably, Stxs are not only potent inducers of cell death, but also activate innate immune responses that may lead to inflammation, and these effects may increase the severity of organ injury in patients infected with Stx-producing bacteria. In the intestines, kidneys, and central nervous system, excessive or uncontrolled host innate and cellular immune responses triggered by Stxs may result in sensitization of cells to toxin mediated damage, leading to immunopathology and increased morbidity and mortality in animal models (including primates) and human patients. Here, we review studies describing Stx-induced innate immune responses that may be associated with tissue damage, inflammation, and complement activation. We speculate on how these processes may contribute to immunopathological responses to the toxins.

## 1. Introduction

Due to the successful application of antibiotics and effective public health measures, many have erroneously come to believe that epidemics of bacterial infectious diseases are no longer a serious risk. However, health threats from Shiga toxin-producing *Shigella* species, especially *Shigella dysenteriae* serotype 1, and *E. coli* continue to pose a threat to populations around the world [1]. Shiga toxin (Stx) was first characterized as an exotoxin synthesized by the bacteria *Shigella dysenteriae* serotype 1. Structurally and functionally related toxins are produced by many serotypes of *E. coli* [2]. 

Shigellosis or bacillary dysentery due to *Shigella* infection continues to be a significant cause of morbidity and mortality in regions where drinking water supplies are contaminated with waste or where human feces is used to fertilize crops consumed by humans. Bacillary dysentery, an acute infectious diarrhea, is a disease of childhood that primarily affects children under 5 years of age [3]. Endemic bacillary dysentery occurs globally, including in portions of Africa, Southeast Asia, and the Indian subcontinent, with estimated incidences as high as 2–7 cases of *Shigella*/1000 children/year requiring clinical care, and 164,300 annual deaths attributable to bacillary dysentery [4]. Although Stx expression was initially characterized in *S. dysenteriae* serotype 1, Stx-producing *S. dysenteriae* of different serotypes [5], and toxin-producing *S. sonnei*, and *S. flexneri* strains have been isolated from humans [6,7,8,9,10]. In contrast to bacillary dysentery, hemorrhagic colitis or bloody diarrhea caused by Shiga toxin-producing *E. coli* (STEC) is more prevalent in developed countries where efficient nation-wide food distribution services allow citizens to consume higher levels of processed foods such as beef and beef products. Although foods containing undercooked contaminated ground beef are common vehicles for outbreaks of bloody diarrhea, other foods have been implicated in both sporadic cases and outbreaks of bloody diarrhea, including unpasteurized fruit juices, sausages, and unwashed vegetables [11,12]. The capacity of STEC to cause widespread outbreaks was highlighted by the outbreak of hemorrhagic colitis caused by Stx-producing *E.coli* O157:H7 focused in and around Sakai City, Japan, in 1996 [13]. In this outbreak, children were fed school lunches containing contaminated white radish sprouts, resulting in over 7,000 infections and approximately 1000 hospitalizations.

Over 200 different STEC O:H-antigen serotypes have been isolated from patients with bloody diarrhea [14]. These organisms have received considerable attention as emergent pathogens due to their capacity to express multiple variants of Stx. The toxins are virulence factors associated with the pathogenesis of bloody diarrhea. However, patients with prodromal diarrheal disease may progress to develop acute renal failure. This renal disease, referred to as diarrhea-associated hemolytic uremic syndrome (D+HUS), is the leading cause of pediatric acute renal failure in the United States [15]. On histopathological examination, the glomeruli of D+HUS patients exhibit extensive swelling and death of microvascular endothelial cells, accompanied by detachment of the cells from the glomerular basement membrane. As a clinical entity, D+HUS is defined by: (i) oliguria progressing to anuria; (ii) hemolytic anemia and schistocytosis (the presence of fragmented and deformed erythrocytes in the bloodstream); (iii) thrombocytopenia (reduction in the abundance of circulating platelets); and (iv) thrombotic microangiopathy (deposition of fibrin-enriched microthrombi within glomerular capillaries and renal arterioles) [16,17,18,19]. 

Several lines of in vivo and in vitro evidence suggest that Stxs and inflammatory mediators induced by the toxins contribute to pathogenesis, in part, by rendering blood vessels in the kidneys, and central nervous system (CNS) more susceptible to the destructive action of Stxs reviewed in ref. [20]. When stimulated with these toxins, intestinal and renal epithelial cells, and intestinal and renal glomerular microvascular endothelial cells, may express neutrophil and monocyte chemoattractants. Studies in animals treated with purified Stxs suggest that peripheral blood mononuclear cells infiltrate the lamina propria and kidneys [20,21,22]. Taken together, these observations suggest that a cytokine- and chemokine-mediated amplification cycle may be initiated in which epithelial and endothelial cells exposed to Stxs produce cytokines and chemokines in a localized manner that facilitate the infiltration of inflammatory cells into sites of toxin-induced damage. These inflammatory cells, in turn, may exacerbate vascular damage through multiple immunopathological mechanisms [23,24]. 

A significant body of research focused on characterizing Stx-induced host signaling pathways has provided strong evidence that the toxins promote ribotoxic and ER stress pathways, leading to inflammation, autophagy and apoptosis reviewed in ref. [25]. However, the precise mechanisms by which Stxs initiate immunopathology remain to be fully characterized. The integration of knowledge on: (i) innate immune responses elicited by the toxins (including activation of complement); (ii) Stx-induced cell stress responses demonstrated in multiple cell types in vitro; and (iii) the pathogenesis of D+HUS caused by the toxins in patients or animal models reviewed in refs. [22,25,26] will be needed to devise novel and effective interventional therapies to prevent or treat the life-threatening complications caused by Stx-producing bacteria. In this review, we will describe our current understanding of immunopathological processes in Stx-induced pathogenesis and the roles of the toxins in initiating these events.

## 2. The Toxins

**Figure 1:**
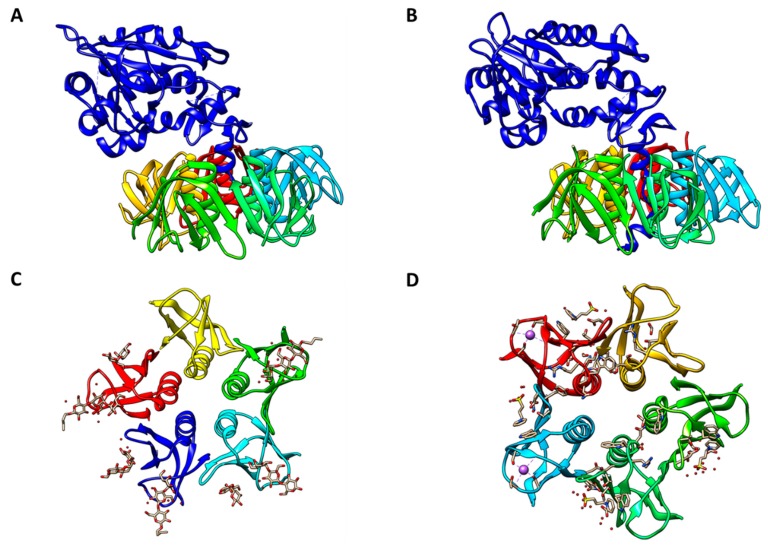
Crystal Structure of Shiga toxins. A. Shiga toxin subtype 1a holotoxin (PDB #1DM0). B. Shiga toxin subtype 2a holotoxin (PDB # 1R4P). C. Shiga toxin subtype 1a B-subunits depicted with potential Gb3 receptor interactions (PDB #1BOS). D. Shiga toxin subtype 2a B-subunits depicted with potential Gb3 receptor interactions (PDB #1R4P, deletion of A-subunit). Toxin A-subunits are shown in dark blue in panels A and B. Individual B-subunits are shown in different colors in all panels. PDB files of all structures were obtained from RCSB PDB (www.rcsb.org) and compile PDB files with Chimera 1.10.2 (UCSF Chimera, www.cgl.ucsf.edu/chimera). Reproduced from reference [25]. 2016, MDPI.

Proteins belonging to the Stx family adopt an AB_5_ molecular configuration consisting of a single A-subunit of ~32 kDa in noncovalent association with a homopentamer of 7.7-kDa B-subunits [27,28] [Figure 1]. Stx enzymatic activity is associated with the A-subunit, while toxin binding to glycolipid receptors maps to the B-subunits. The B-subunit homopentamer possesses 15 potential receptor binding sites, suggesting that Stxs may effectively cross-link membrane glycolipids on target cells [29,30,31]. The prototypical member of the Stx family is Shiga toxin expressed by *Shigella dysenteriae* serotype 1. Early studies to characterize STEC toxins revealed two related Shiga toxin types: Shiga toxin type 1 (Stx1) which is essentially identical to Shiga toxin expressed by *S. dysenteriae* serotype 1, and the more genetically variant Shiga toxin type 2 (Stx2) [32]. The two major toxin types are sufficiently antigenically distinct that polyclonal antisera raised against one toxin type fails to neutralize cytotoxicity of the heterologous toxin type [33]. Over the next three decades, genetic variants of Stx1 were characterized into toxin subtypes Stx1a, Stx1c and Stx1d, and those of Stx2 into Stx2a, Stx2b, Stx2c, Stx2d, Stx2e, Stx2f and Stx2g reviewed in refs. [34,35]. Overall, Shiga toxin type 1, now designated Stx1a, shares only 56% amino acid identity with Shiga toxin type 2, now Stx2a, although residues involved in enzymatic activity and binding to cells are more highly conserved. In contrast, variation within the Stx1 and Stx2 subtypes is much lower, with members sharing 84%–99% amino acid identity. The Stx2 subtypes differ from one another to a greater extent than those of the Stx1 subtypes. Operons for the A- and B-subunits of Stx1 and Stx2 subtypes are encoded by lambdoid bacteriophages. Thus, STEC are lysogens containing one or more toxin-converting prophages. Although mosaicism is common in toxin-converting lambdoid phages characterized so far, the toxin operons usually map immediately downstream of the phage P_R_ promoter and transcription antiterminator Q. Consequently, the signals that induce the lytic cycle, such as ultraviolet light or DNA damaging drugs, also induce expression of the toxin operons [36,37,38].

Toxin subtype differences are associated with different severity of disease in humans, and different levels of toxicity in animal models of disease [22,39,40,41,42]. Stxs were initially shown to bind with high affinity to the glycolipid Gal(α1→4)Gal(β1→4)Glc(β1→1)ceramide (globotriaosylceramide or Gb3) on host cell membranes, acting as a toxin receptor [29,43,44,45]. 

The toxin subtypes have recently been shown to have different receptor binding preferences [46,47]. For example, Stx1a preferentially binds Gb3 with detectable binding to globotetraosylceramide (Gb4), while Stx2a shows a strong preference for Gb3 and marginal binding to Gb4. Toxin subtype Stx2e displays a more promiscuous pattern of glycolipid binding, with preferential binding to Gb4, but also binding Gb3, pentahexosylceramides with Gb4-elongated core structures, including Forssman glycosphingolipid, and globopentaosylceramide (Gb5) [48,49]. Legros and colleagues [47] characterized the Stx-binding glycolipids expressed in detergent-resistant membrane preparations from Madin-Darby canine kidney (MDCK) II epithelial cells. The Stx1a-, Stx2a- and Stx2e-binding glycolipids Gb3 and Gb4, and the Stx2e-binding Forssman glycosphingolipid, were expressed by the cells. Surprisingly, MDCK II cells were refractory to cytotoxicity mediated by Stx1a, Stx2a and Stx2e. The resistance to Stx cytotoxicity correlated with the absence of plasma membrane localization of the toxin binding glycolipids as determined by immunofluorescence confocal laser scanning microscopy. Thus, the detection of Stx receptors in detergent-resistant membrane preparations alone is not sufficient to predict sensitivity to the cytotoxic action of Stxs.

The precise mechanisms responsible for toxin resistance in MDCK II cells remain to be defined, but it should be noted that the availability of glycolipids to serve as receptors is affected by alterations in the lipid component of the glycolipids and the membrane microenvironment. Changes in the lipid moiety of glycolipids that may modulate receptor function include fatty acid heterogeneity, chain length, degree of saturation and fatty acid hydroxylation [50,51,52]. Differences in glycolipid acyl chains may affect lipid bilayer properties. For example, the capacity of Stx B-subunits to form tubular invaginations at the cell surface appears to require unsaturated Gb3. Furthermore, Gb3 with long acyl chains may cross the lipid bilayer to cause localized bilayer rigidity reviewed in ref. [53]. Variability in the lipid moiety may alter the membrane microenvironment so that the conformation of the disaccharide binding domain of Stx-binding glycolipids is changed and receptor-ligand interactions are disrupted [53]. Finally, in vitro studies have shown that the accumulation of toxin binding glycolipids in cholesterol enriched cell membrane domains may result in “masking” of the receptors and interference with receptor signaling and internalization functions [47,54,55]. 

The binding of toxin B-subunits initiates uptake of the toxins into target cells [56]. Once the toxin is bound to its receptor, it may be endocytosed utilizing clathrin-coated pits into an early endosome [57]. However, studies designed to block clathrin function only partially inhibited Stx uptake [58,59], suggesting that clathrin-independent pathways may also function in toxin internalization. Several recent studies have begun to characterize clathrin-independent uptake mechanisms involving toxin clustering at the plasma membrane, resulting in inward-oriented membrane curvature, and the formation of membrane invaginations and tubular endocytic pits [60,61,62]. However, it is important to note that while there may be alternative clathrin-independent mechanisms of Stx endocytosis, clathrin and its associated protein epsinR have been shown to be essential for proper trafficking of internalized Stxs from early/recycling endosomes to the *trans*-Golgi network in HeLa cells [59]. 

Once internalized, the toxins are subsequently routed to the *trans*-Golgi network, through the stacks of the Golgi apparatus, and into the ER lumen; a process termed retrograde transport [63]. Retrograde transport of Stxs has been shown to be dependent on a large number of intracellular trafficking proteins reviewed in ref. [60]. Each of the toxin transport steps between intracellular organelles represents a target for the development of inhibitors to block cell intoxication. Cleavage of the A-subunit into the A1 and A2 fragments is thought to occur through the enzymatic action of furin during toxin trafficking [64]. The A1 and A2 fragments remain linked by a disulfide bond between cysteine residues C242 and C261. In the ER, the disulfide bond is broken and the A1 fragment retrotranslocates across the ER membrane to enter the cytoplasm [65,66]. 

The A1 fragment of the toxin has RNA *N*-glycosidase activity and inactivates the 60S subunit of host cell ribosomes by cleaving the *N*-glycosidic bond of a single adenine residue in 28S rRNA (A4324 in rats). The target adenine attacked by Stxs is located within a highly conserved structure referred to as the α-sarcin/ricin loop [67]. This site is the target for many ribosome inactivating proteins reviewed in refs. [68,69]. The Stx-mediated depurination reaction leads to inhibition of elongation factor 1-dependent aminoacyl-tRNA binding, and inhibition of protein synthesis [70]. Retrotranslocation appears to involve the transient unfolding of the A1 fragment and interaction of a hydrophobic domain within the A1 fragment with the Sec61 translocon-associated ER luminal chaperones HEDJ, BiP and GRP94 [66,71]. The transient unfolded form of the toxin may be required for the activation of host cell stress response pathways involved in cytokine production, autophagy and apoptosis. Once in the cytoplasm, the A1 fragment re-folds and in some way evades ubiquitination and destruction by the ER-associated protein degradation (ERAD) pathway. The observation that many subunit toxins (Stxs, cholera toxin, ricin) utilize the ERAD system to access the cytoplasm raises the possibility that enzymatically inactive holotoxoids may be developed and used as translocon competitors to treat genetic diseases, such as cystic fibrosis, that involve protein misfolding [72].

Although protein synthesis inhibition may lead to cell death, we and others have investigated multiple biological functions of Stxs in vitro and in vivo. The data suggest that Stxs may cause cell death by multiple pathways, including apoptosis and autophagy. Over the course of these studies, investigators have identified and devised approaches for interrupting Stx-induced host signaling mechanisms such as ribotoxic and ER stress signaling reviewed in ref. [25]. Disruption of Stx-induced signaling cascades may provide targets for the development of inhibitors of the cytotoxic action of the toxins. 

## 3. The Role of Stxs in Innate Immunity

Macrophages are critical sentinel cells programed to dramatically up-regulate cytokine expression upon encounter with microbial products such as Stxs in the gut, the bloodstream, and within target organs. Macrophages respond to microbial infections by regulating inflammation and innate immunity in complex ways. For example, upon engagement of pattern recognition receptors, macrophages dramatically alter their transcriptional profile and produce cytokines that initiate inflammation [73]. Macrophages may be activated by cytokines to achieve an enhanced microbicidal or tumoricidal capacity [74]. Macrophages engage in efferocytosis, the clearance of apoptotic neutrophils and cellular debris that may result from tissue damage caused by microbial toxins, and in the process trigger the resolution of inflammation and initiation of wound repair [75]. Cytokines play essential autocrine and paracrine signaling roles in all these processes. In vitro studies revealed that Stxs regulate signaling pathways involved in expression of cytokines and chemokines by human and murine macrophages, as well as macrophage-like cell lines, at the transcriptional and post-transcriptional levels [76,77,78]. Clinically relevant target cells of the immunomodulatory activities of Stxs appear to include neutrophils and monocytes, while renal and CNS microvascular endothelial cells and renal tubular epithelial cells appear to be primary targets for the cytotoxic action of the toxins reviewed in ref. [79]. However, all of these cell types may be capable of expressing cytokines upon interaction with the toxins. 

**Figure 2:**
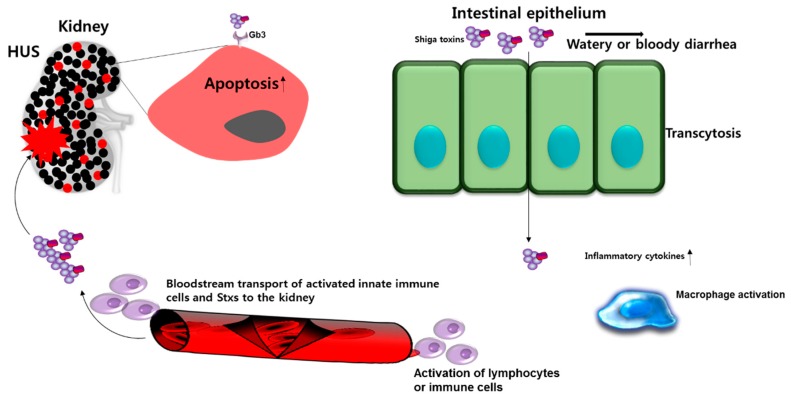
Shiga toxin-induced immunopathology. Shiga toxins may cross the intestinal epithelial cell barrier by mechanisms involving M-cell uptake, transcellular transport, or paracellular transport. Once in the submucosa, the toxins may directly damage the intestinal microvasculature and elicit cytokine and chemokine production by resident macrophages. Macrophage activation results in the infiltration of neutrophils and monocytes which may further exacerbate tissue damage. Neutrophils and monocytes may also act as “carrier” cells to transport toxins in the bloodstream. Once in microvessels, such as glomeruli, that are rich in the toxin receptor, Gb3, the toxins may be transferred from the carrier cells to damage glomerular endothelial cells and tubular epithelial cells. The localized production of cytokines may up-regulate the expression of toxin receptors on some cell types, and activation of the complement cascade may further damage target organs.

The pathophysiological consequences of activation of innate immunity by Stxs may include: i) changes in cell morphology and intercellular tight junctions in the intestinal epithelial barrier that allow Stxs to cross into the lamina propria where the toxins damage colonic blood vessels and initiate hematogenous spread; ii) the facilitation of chemotactic infiltration of inflammatory cells into the lamina propria of the gut and into the kidneys; and iii) the up-regulated expression of Gb3 on microvascular endothelial cells [76,77,78,79,80,81,82]. In D+HUS patients infected with STEC, elevated, localized expression of pro-inflammatory cytokines and chemokines by tissue or infiltrating macrophages may accelerate disease progression (Figure 2). We reported that the innate immune response induced in macrophage-like cell lines by Stxs also includes components responsible for down-regulation of inflammation, i.e., the toxins appear capable of triggering a balanced pro- and anti-inflammatory response in vitro [78,83]. Numerous studies in animals, as well as clinical studies in humans, showed that renal proximal tubule epithelial cells are targeted for destruction by Stxs, and the localized production of cytokines by renal cells may exacerbate tubular damage [84,85,86,87,88,89,90]. Differences in the capacity to regulate cytokine/chemokine expression during the course of the innate immune response, as well as differences in the ability to induce cell death may explain the greater potential of Stx2a to cause extra-intestinal complications such as D+HUS and CNS abnormalities, and fatality in humans.

## 4. Role of Stxs in Gut Immunopathogenesis

In contrast to our detailed understanding of the biochemical properties of Stxs, we have yet to elucidate the precise roles of these toxins in the pathogenesis of the prodromal diarrheal disease that may precede systemic complications. The major pathogenic determinant of *Shigella* species is their invasiveness [91,92]. Specifically, these organisms are capable of invading colonic epithelial cells through basolateral membranes by a mechanism referred to as parasite-directed phagocytosis. Once inside host cells, the bacteria rapidly lyse the endosome and replicate within the cytoplasm. The bacteria can move within cells by a process that involves nucleation of actin at a single bacterial pole. In this manner, *Shigella* may propel themselves into adjacent, non-infected colonic epithelial cells. 

In a seminal study reported in 1988, Sansonetti and colleagues fed a Shiga toxin-producing wildtype *S. dysenteriae* serotype 1 strain (Tox^+^) or its isogenic atoxigenic (Tox^−^) mutant strain to macaque monkeys [93]. All the animals developed fulminant dysentery, highlighting the importance of the invasive phenotype in the pathogenesis of shigellosis. However, blood was noted only in the stools of animals that received the Tox^+^ strain. Histopathologic examination of animals infected with Tox^+^ or Tox^−^ bacteria revealed significant differences. Infection with the toxin-producing strain was correlated with a profound reduction in numbers of circulating neutrophils, injury to colonic capillaries within the lamina propria, and perivascular inflammation involving blood vessels of the peritoneal mesothelium. These changes were not observed in animals receiving the Tox^‒^ strain. Thus, bacterial invasion and ulceration occurred independently of toxin synthesis, while the production of Shiga toxin was associated with increased colonic vascular damage and leukocyte extravasation and infiltration into the colonic mucosa. 

In contrast to *Shigella*, STEC are not invasive, but they are capable of colonizing the human intestinal tract. The organisms appear to first colonize the distal ileum followed by the colon [94,95]. Initial adherence of STEC may be mediated by a type IV pilus referred to as hemorrhagic coli pilus [96]. A subset of STEC, referred to as enterohemorrhagic *E. coli* (EHEC), express genes that allow the bacteria to adhere to the gut in an intimate manner [97,98]. Following initial adherence, these close interactions at epithelial surfaces, termed attaching and effacing (A/E) lesions, result in the effacement of the normal brush border, which is replaced by a single “pedestal” subjacent to the adherent bacteria [99,100]. The formation of A/E lesions, and expression of the full range of EHEC virulence determinants, are dependent on a type III secretion system (T3SS) that translocates as many as 39 effector proteins from the bacteria into the host cell cytoplasm reviewed in ref. [101]. Recently, the Sperandio laboratory has demonstrated that neurotransmitter signaling through epinephrine and norepinephrine within the gut is crucial for the noninvasive enteric pathogens EHEC and *Citrobacter rodentium* to fully activate virulence mechanisms, including actin polymerization and A/E lesion formation during gastrointestinal infection [102,103]. Although the drastic molecular reconfiguration of the epithelial cell apical surface associated with A/E lesion formation contributes to EHEC adherence and the development of diarrhea [104,105], the adherent bacteria also produce Stxs. Once translocated into the lamina propria, Stxs induce expression of chemokines by intestinal epithelial cells, leading to increased leukocyte extravasation and infiltration into the intestinal mucosa [106,107,108,109,110,111]. According to an opinion piece by Heyderman et al. [112], Stxs expressed by STEC in the gut may activate immune cells, particularly inflammatory T cell subsets, presumably through the action of intestinal epithelial cell-secreted immunomodulatory molecules. The presence of activated T-cell subsets in the circulation may contribute to disease progression from the diarrheal phase to D+HUS. 

As mentioned above, in order to spread through the bloodstream and cause systemic disease, Stxs must first cross the intestinal epithelial barrier. Precisely how this occurs remains to be fully understood and is the focus of intense experimental scrutiny. The observation that EHEC serotype O157:H7 preferentially associated with follicle-associate epithelium [94,95] raised the potential that STEC may interact with microfold cells or M-cells, specialized epithelial cells capable of translocating antigens “sampled” from the intestinal lumen to the basolateral side of the epithelial barrier [113]. Once delivered to the gut-associated lymphoid follicle, the sampled antigen interacts with macrophages and dendritic cells, i.e., cells capable of processing and presenting antigens for activation of adaptive immunity. Recently, Etienne-Mesmin and colleagues [114] used murine Peyer’s patches and a human M-cell in vitro system to show that EHEC O157:H7 strains are translocated across these mucosal barriers. The organisms survived uptake by THP-1 macrophage-like cells, replicated and produced Stxs, and then triggered apoptosis of the cells. Thus, a potential mechanism for EHEC delivery across the intestinal epithelial barrier may utilize specialized host cells that sample antigen and activate adaptive immunity. 

Alternatively, rather than using the M-cell “Trojan horse” mechanism of crossing the gut barrier, Stxs may simply knock at the front door. Stx1a was reported to cross polarized T84 epithelial cell monolayers without altering transepithelial resistance [115]. Furthermore, immunoelectron microscopy of toxin treated T84 cells localized Stx1 in endosomes, Golgi, ER and nuclear membrane, but not at tight junctions, suggesting that transcytotic mechanism(s) may be involved in toxin translocation. Macropinocytosis is a clathrin- and caveolin-independent mechanism utilized by intestinal epithelial cells for the internalization of relatively large molecules. Lukyanenko et al. [116] showed that Stxs are associated with actin-coated macropinosomes in intestinal epithelial cells, and once internalized, the toxins were transported from apical surfaces to basolateral surfaces. The critical role of actin rearrangement in formation of A/E lesions raised the possibility that effector molecules delivered through the type III secretion apparatus may be involved in toxin translocation. However, macropinocytosis of Stxs was shown to occur independently of the EHEC T3SS, and the membrane protein intimin, involved in adherence and A/E lesion formation [117]. STEC *E. coli* O104:H4, a serotype associated with a major outbreak of hemorrhagic colitis and D+HUS, does not express intimin, yet still efficiently translocates Stxs using macropinocytosis. In fact, intact STEC bacteria were not necessary to induce macropinocytosis. Rearrangement of F-actin and facilitated transport of Stxs were mediated by soluble factors in bacterial lysates. Interestingly, actin rearrangement events triggered by STEC lysates do not involve cortactin dephosphorylation, an event associated with pedestal formation, nor do the lysates trigger the activation of the non-receptor tyrosine kinase Src, an event associated with actin remodeling in epithelial cells. In et al. [117] showed that the secreted serine protease EspP, and possibly other serine proteases, appear to be critical for the enhanced toxin uptake and actin remodeling associated with macropinocytosis. Thus, toxin transport in intestinal epithelial cells may utilize a previously unrecognized signaling mechanism for actin rearrangement activated by the presence of STEC at the apical surface and leading to toxin transport across the intestinal epithelial barrier. 

Finally, Stxs may slip in through the side alley. Hurley et al. [118] reported that human neutrophils were able to transmigrate across a polarized T84 intestinal epithelial monolayer. The basolateral to apical transmigration of neutrophils was associated with the increased translocation of Stx1a and Stx2a in the opposite direction. Toxin translocation was correlated with the movement of [^3^H]-inulin across the monolayer, suggesting a paracellular transport mechanism. Thus, the infiltration of neutrophils into the submucosa and transmigration into the intestinal lumen may facilitate paracellular transport of the toxins across the intestinal epithelial cell barrier. Another consideration in the study of Stx paracellular translocation is the role of virulence factors other than the toxins. For example, EspF_U_, an EHEC effector protein delivered into host cells by the T3SS, causes extensive changes in the phosphorylation and distribution of intercellular tight junction proteins, leading to reduced transepithelial electrical resistance [119]. These changes may be sufficient to facilitate paracellular transport of Stxs.

An important consideration in interpreting toxin translocation studies in vitro is the expression of the toxin receptor. Many studies have utilized the T84 epithelial cell line which, in the differentiated state, expresses scant Gb3 and is resistant to Stx-mediated protein synthesis inhibition [120]. In contrast, many other transformed intestinal epithelial cell lines express membrane Gb3 and are sensitive to Stx cytotoxicity [121,122]. Normal human intestinal cells have been reported to express no or scant Gb3, although the alternative, lower affinity toxin receptor Gb4 appears to be more abundant on human colonic tissue sections [120,123]. In contrast to intestinal epithelial cells, Paneth cells, a small intestinal crypt cell involved in the production of antimicrobial products such as defensins, has been shown to express Gb3 and bind Stx1a and Stx2a [124]. Additional studies will be needed to determine whether Paneth cells play a prominent role in toxin translocation. 

In summary, the precise mechanism(s) of Stx translocation across the intestinal epithelial barrier remain to be fully characterized, but the data reported to date suggest that multiple pathways may be involved, perhaps operational at different locations in the intestinal tract, and under different environmental conditions. Furthermore, toxin translocation across the gut epithelial barrier may include Gb3-dependent and Gb3-independent processes.

Studies on how T3SS effectors affect toxin internalization and routing in epithelial cells expressing Gb3 are ongoing. Pacheco and colleagues [125] performed a genome-wide clustered regularly interspaced short palindromic repeats with Cas9 (CRISPR/Cas9) loss-of-function screening in the colonic epithelial cell line HT-29 to identify mutants with elevated rates of survival following infection with EHEC O157:H7 strain EDL933 Δ*espZ.* This strain has high levels of T3SS activity and produces both Stx1a and Stx2a. The screen identified three categories of genetic loci that conferred survival following infection: genes encoding enzymes involved in sphingolipid or glycosphingolipid biosynthesis, including the terminal steps in Gb3 synthesis; genes associated with cell proliferation; and two genes encoding poorly characterized proteins, lysosomal associated protein transmembrane 4 alpha (LAPTM4A) and transmembrane 9 superfamily member 2 (TM9SF2). The use of EHEC deletion mutants with deficiencies in T3SS (Δ*espZ* Δ*escN*) or toxin production (Δ*stx1* Δ*stx2*) revealed that the identified genes conferred resistance to both translocated effector molecules and Stxs. As mentioned earlier, the T3SS-mediated translocation of EHEC effectors is essential for the extensive reconfiguration of host cell actin and other cytoskeletal elements that leads to pedestal formation [126,127]. Interestingly, mutants in glycosphingolipid synthesis, as well as LAPTM4A and TM9SF2 mutants, showed reduced translocation of the effector molecule translocated intimin receptor (Tir), a reduced percentage of infected cells with pedestals, and a reduced number of pedestals per cell [125]. Both the Gb3 synthesis mutants and the LAPTM4A and TM9SF2 mutant cells bound lower levels of fluorescently tagged Stxs, even when the cells were permeabilized to detect toxin binding to intracellular receptors. Confocal microscopy revealed that LAPTM4A and TM9SF2 localize to the Golgi apparatus. Thus, glycosphingolipid synthesis and/or intracellular trafficking may require functional LAPTM4A and TM9SF2 activity. 

Tian et al. [128] performed an independent genome-wide CRISPR/Cas9 loss-of-function screening of the human bladder carcinoma cell line 5637 to identify host factors important in intoxication by Stx1a, Stx2a and ricin. This screen identified LAPTMA4 as being selectively required for Stx intoxication while TM9SF2 was required for both Stx and ricin intoxication. The screen also identified the Golgi-localized protein transmembrane protein 165 (TMEM165) as necessary for Stx and ricin intoxication. Biochemical characterization of the roles of these intracellular proteins suggested that LAPTM4A is a critical cofactor for glycosyltransferase activity in Gb3 biosynthesis. TMEM165 has been reported to be important in Mn^2+^ homeostasis, which serves as a co-factor in the Golgi for glycosyltransferases involved in building glycoproteins and glycolipids [129]. Tian et al. [128] showed that Mn^2+^ supplementation of TMEM165 KO cells restored Stx binding. The function of TM9SF2 remains poorly characterized. Taken together, these data suggest that genetic loci involved in Gb3 synthesis and intracellular trafficking impact both Stx binding and susceptibility to T3SS effector activity. From a therapeutic standpoint, the CRISPR loss-of-function screening studies are clinically important because they identify early steps in pathogenesis. The results suggest that agents designed to inhibit Gb3 synthesis and intracellular routing could protect Gb3-expressing cells against EHEC infection by disrupting T3SS effector delivery and Stx intoxication. 

During the prodromal diarrheal phase of disease, Stxs may induce expression of a broad array of pro-inflammatory molecules by intestinal epithelial cells, as well as endothelial cells and macrophages present in the lamina propria. Among these mediators, some (TNFα and IL-1β) may exacerbate vascular damage through sensitization of endothelial cells in the gut and other organs by up-regulating the expression of the toxin receptor Gb3 [130,131,132,133]. However, human intestinal microvascular endothelial cells have been reported to constitutively express high levels of Gb3 [134] suggesting that cytokine-mediated sensitization may be less critical for vascular damage in the gut. 

The presence of the toxins and the cytokines they elicit may also drive monocyte differentiation or activation. Using the monocytic cell line THP-1, we found that undifferentiated monocytic cells were relatively poor producers of cytokines when exposed to Stxs, and that the cells were relatively sensitive to killing by the toxins [135]. Differentiated, plastic-adherent macrophage-like THP-1 cells produced soluble cytokines when stimulated with toxins but were also sensitive to induction of programmed cell death [135,136]. Thus, induction to an activated state by Stxs, or by other microbial products such as endotoxins, may facilitate cytokine production in macrophages, but ultimately cause the cells to undergo apoptosis. In addition, activation of leukocytes has been shown to increase microvesicle production and release [137]. Stxs stimulate macrophages to release an inducer of granulocyte hematopoiesis, G-CSF, as well as the chemokines CCL2, CCL4, and CXCL8, which together may trigger neutrophilia and neutrophil infiltration into intestinal sites of damage. The presence of neutrophils in the intestinal mucosa may cause direct tissue damage but may also be important for hematogenous toxin transport to the kidneys and other target organs. Stxs may transiently bind or “piggy back” on neutrophils and monocytes [138,139], and/or bind neutrophil- and monocyte-derived microvesicles for intravascular transport of the toxins to distal sites of organ damage [137,140]. Hematogenous delivery of the toxins to target organs is discussed in more detail below. 

## 5. The Role of Stxs in Renal Immunopathogenesis

A series of events must occur to lead to the development of D+HUS. As noted above, STEC initially bind to intestinal epithelial cells, followed by M-cell directed transport of toxin-producing organisms to lymphoid follicle-associated antigen presenting cells and/or transcellular or paracellular transport of toxin molecules across the intestinal epithelium [141]. Once within the lamina propria, the toxins trigger vascular damage and inflammation, “calling in” the cells involved in tissue damage and hematogenous spread of the toxins.

Histopathological studies suggest that once Stxs enter the bloodstream, they target blood vessels for destruction in specific organs. Several lines of in vitro and in vivo evidence related to D+HUS pathogenesis indicate that the kidneys are the primary target organs in which Stxs cause acute dysfunction. In particular, renal glomerular endothelial cells and proximal tubule epithelial cells are clinically relevant target cells damaged by the toxins in the kidney. However, endothelial and epithelial cells may also respond to Stxs by localized production of pro-inflammatory cytokines [142,143]. The direct cytotoxic action of Stxs on the glomerular microvasculature and collecting tubules, and the localized production of cytokines within these renal compartments may be necessary for the development of D+HUS [144]. D+HUS patients frequently have elevated levels of urinary and plasma cytokines and chemokines reviewed in ref. [145]. However, additional studies are necessary to define the precise roles of pro-inflammatory cytokines in D+HUS pathogenesis.

The timing of cytokine production versus exposure to Stxs may be critical for the disease outcome. Mice injected with recombinant TNF-α using the intraperitoneal (i.p.) route 1 h prior to toxin challenge were protected from lethality, whereas mice injected with TNF-α after the toxin, exhibited accelerated lethality associated with more severe glomerular damage [146]. In baboons treated with Stx1a or Stx2a, the two toxins caused glomerular and tubulointerstitial injury and induced leukocyte chemotactic responses in the kidneys; these responses, along with an elevated number of eosinophils in renal inflammatory cell infiltrates following Stx2a challenge, contributed to pathology [147]. Although renal infiltration by eosinophils is a well-characterized predictor of severe kidney failure in patients with autoimmune anti-neutrophil cytoplasmic antibody (ANCA)-associated vasculitis [148], the pathogenic role of eosinophils in the kidneys of patients with D+HUS remains to be determined. Stearns-Kurosawa et al. [147] reported that chemokine mRNA expression in baboon kidney tissue, and urine levels of chemokines (particularly IL-8, MCP-1, and MIP-1α), were markedly elevated following administration of a high dose of Stx1a or Stx2a. Thus, the establishment of this renal chemotactic milieu may lead to a further influx of leukocytes to exacerbate tissue damage, and in some instances, the infiltrating cells may be toxin carriers for further toxin deposition in the renal microvasculature. Patients with D+HUS may be endotoxemic [149,150] and endotoxins (lipopolysaccharides) are known to be powerful inducers of the innate immune response, including pro-inflammatory cytokine expression [151]. It is not yet completely understood how Stxs (with or without endotoxemia) regulate intracellular events to augment pro-inflammatory cytokine or chemokine expression in the renal glomerular and collecting tubule compartments during the progression to D+HUS. However, several studies have examined the capacity of Stxs to activate transcriptional factors such as NF-κB and AP-1 known to regulate cytokine and chemokine gene expression [152,153,154]. Stxs appear to regulate gene expression through activation of multiple mitogen-activated protein kinases and tyrosine kinases reviewed in ref. [155]. Finally, Ramos et al. [156] reported that dysregulation of chemokine receptors in circulating monocytes recruited to injured kidneys may be associated with the severity of D+HUS. 

A complete compendium of risk factors leading to the development of D+HUS remains to be definitively established. Not all patients with bloody diarrhea caused by STEC progress to acute renal failure. It is estimated that 5%-15% of patients with bloody diarrhea will develop HUS [18]. Patients infected with STEC strains capable of expressing Stx2a, either alone or in combination with other Stx subtypes, are at increased risk for progression from diarrheal disease to renal (and CNS) complications [39,40,157]. D+HUS associated with STEC infection is primarily a disease of children <5 years of age, although the elderly are also at increased risk for developing HUS. The use of antibiotics is contraindicated during the prodromal diarrheal phase of disease, since antibiotic use is associated with an increased risk of progression to D+HUS [158]. As emphasized by Tarr et al. [159], how one clinically defines D+HUS is critically important in any study examining risk factors. In addition to monitoring urine output, blood dyscrasias (erythrocyte and platelet counts) and coagulopathies, blood urea nitrogen (BUN) and serum creatinine levels are crucial indicators of kidney dysfunction in D+HUS patients, as well as in animal models challenged with Stxs [160].

Initially, *E. coli* O157:H7 was the predominant serotype isolated from D+HUS patients in many parts of the world. Recently, however, non-O157:H7 serotypes, including O26, O111, O121, O145, O91, O103, and O80, have been increasingly isolated from D+HUS patients. A recent outbreak of bloody diarrhea focused in northern Germany, caused by ingestion of bean sprouts contaminated with *E. coli* O104:H4, introduced some troubling new aspects of concern. First, the *E. coli* O104:H4 serotype was the initial enteroaggregative *E. coli* to be characterized as an STEC associated with a major outbreak of food-borne illnesses. The strain had acquired the genes encoding Stx2a. Second, the vast majority (88%) of cases with extra-intestinal complications such as D+HUS and CNS disease were manifest in previously healthy adults. Only 2% of D+HUS cases were children < 5 years of age. Third, the incidence of D+HUS and CNS disease following hemorrhagic colitis caused by *E. coli* O104:H4 was on the order of 22% in adults [161,162,163,164]. Multi-year epidemiologic studies of outbreaks caused by EHEC revealed gender differences in D+HUS incidence, with cases predominantly seen in women [165,166]. In an outbreak caused by *E. coli* O157:H7 in China in 1999, 62.1% of cases were women, and in the recent outbreak caused by *E. coli* O104:H4 in Germany, 58% of cases of prodromal diarrheal disease were women, and 68% of cases progressing to D+HUS were women [162,167]. Over the past few decades, it has become apparent that obese patients (representing a significant proportion of the U.S. population) have increased susceptibility to numerous diseases, including infectious diseases. More recently, Harrison et al. [168] established a diet-induced obesity (DIO) mouse model to evaluate the impact of DIO on susceptibility to D+HUS pathogenesis. Following exposure to multiple sub-lethal doses of Stx2a, both toxin-treated wild-type (WT) and DIO mouse kidneys had significantly higher degrees of renal tubular dilation and necrosis and pro-inflammatory responses than saline controls; however, no differences in histopathology or cytokine responses were detected between toxin-treated WT and DIO mice. 

As mentioned earlier, Stxs do not appear to circulate freely in the bloodstream, but instead bind to multiple types of blood cells, including neutrophils [138], monocytes [169], platelets [170,171], and erythrocytes [172,173]. These cells may serve as carriers to transport toxins to microvascular endothelial cells that express high levels of the toxin receptor Gb3, such as glomerular capillaries and the microvasculature of the CNS [79]. Direct cytotoxic action of the toxins, or exacerbation of toxin damage following localized cytokine/chemokine expression, results in swelling and detachment of endothelial cells from the basement membrane, which is the histopathological hallmark of D+HUS. Microvascular damage is followed by deposition of fibrin and platelet thrombi, and collectively, these changes are referred to as thrombotic microangiopathy. 

In 1980, Monnens and colleagues [174] detected elevated levels of C3 and Factor B breakdown products in the sera of children with D+HUS, suggesting that dysregulation of the alternative pathway of complement activation may be a factor in D+HUS pathophysiology [175]. Morigi and colleagues [176] used immunofluorescence microscopy to show that HMEC-1 dermal endothelial cells treated with Stx1a bound C3 on the cell surface when perfused at high shear stress with 50% human serum as a source of complement. Under these conditions, addition of complement receptor-1 decreased C3 activation on Stx1a-treated endothelial cells, whereas addition of the chelating agent MgCl_2_-EGTA revealed activation of the alternative complement pathway. Treatment of HMEC-1 cells with Stx1a and human serum increased the expression of P-selectin, and Stx1a-mediated C3 deposition co-localized with P-selectin on the surface of the cells. By contrast, Stx1a slightly decreased expression of the antithrombotic molecule thrombomodulin (TM). TM expression was decreased further through generation of the anaphylatoxin C3a, upon perfusion with human serum. Double perfusion of toxin-treated cells with 50% human serum and fluorescently labeled human blood cells revealed extensive thrombus formation on endothelial monolayers [176]. 

These observations were recapitulated in an animal model of D+HUS. Mice given Stx2a + LPS exhibited higher levels of P-selectin expression, TM shedding, and C3 deposition on glomerular endothelial cells, resulting in fibrin(ogen) deposition, platelet clumping, and thrombocytopenia [177]. Moreover, histopathologic changes and C3 deposition in mice administered Stx2a + LPS were not limited to glomerular endothelial cells. The animals exhibited reduced podocyte numbers per glomerulus, as well as reduced podocyte density, within 24 h of Stx2a + LPS injection. Ultrastructural changes included endothelial cell swelling and effacement of podocyte foot processes. Immunofluorescence microscopy revealed enhanced C3 deposition, which co-localized with nephrin staining on podocytes. These changes were not detected on podocytes from mice deficient in Factor B (Bf^−/−^) [177]. Podocyte ultrastructural changes correlated with up-regulated expression of the ankyrin repeat-containing serine/threonine kinase integrin-linked kinase (ILK) and the zinc-finger transcription factor Snail. Snail down-regulates expression of nephrin and epithelial ZO-1, potentially leading to altered intercellular adhesion, slit diaphragm disruption, and proteinuria [178,179,180]. Immunofluorescence microscopy studies confirmed activation of ILK, elevated Snail expression, and reduction in nephrin expression in renal sections from mice treated with Stx2a + LPS. Again, these changes were not seen in Bf^−/−^ mice. Co-treatment with a C3a receptor antagonist reduced podocyte damage and loss, prevented ILK activation, blocked changes in Snail and nephrin expression, and limited renal dysfunction as assessed by thrombocytopenia and serum BUN levels [177]. 

Orth et al. [181] showed that purified Stx2a activated complement directly in the fluid phase, as reflected by elevated formation of terminal complement components (TCC) in normal human serum incubated with the toxin. Stx2a-mediated TCC formation fell markedly in the presence of the chelator EDTA, which blocks both the classical and alternative pathways of activation. By contrast, TCC levels were comparable in sera incubated with Stx2a in the presence or absence of Mg^2+^-EGTA, which specifically blocks classical pathway activation. In addition, Stx2a binds Factor H and related molecules through interactions with multiple short consensus repeats [181,182]. Factor H is an essential cofactor of Factor I-mediated cleavage of C3b to iC3b. Stx2a binding to Factor H does not abolish Factor H activity in the fluid phase but does delay cleavage of the C3b α-chain when C3b is fixed and covalently attached to the surface of CHO cells. In this regard, the ability of Stx2a to bind short consensus repeats located at the carboxy-termini of Factor H may impair the capacity of Factor H to interact with cell surfaces, leading to delayed C3b inactivation and prolonged C3 convertase activity. Collectively, these data suggest that the capacity of Stxs to directly activate the alternative pathway of complement, or to mediate complement fixation on renal cell membranes may contribute to the pathogenesis of D+HUS.

## 6. Roles of Stxs in CNS Immunopathogenesis

The development of neurological injury following hemorrhagic colitis is associated with a poor clinical outcome and is a frequent cause of acute fatality [183]. The actions of Stxs on the microvasculature of the spinal column and the brain are thought to be a major cause of CNS disease. Numerous clinical case reports show that some pediatric patients with D+HUS develop severe neurological manifestations associated with Stx-mediated renal damage [164,184,185,186,187]. STEC O104:H4-infected D+HUS patients developed neurological manifestations, including lethargy and impaired visual function with complete blindness, supporting ocular involvement in D+HUS pathogenesis [164,188]. More recently, Park et al. [189] reported that Stxs activate apoptotic signaling and the ER stress response in human retinal pigment epithelial cells, which are located just posterior to the photoreceptors (a specialized type of neuron in the retina). Notably, the inflammatory response that occurs in response to Stx-mediated vascular injury in severe cerebral edema can lead to seizures, strokes, coma, and death. Reminiscent of the neurologic symptoms reported in patients infected with STEC, non-human primates treated with Stxs suffered seizures and coma, which progressed to death [86,190,191]. In addition to the baboon D+HUS model, animal models mimicking human clinical symptoms have been used as in vivo tools to reveal the molecular basis of Stx-induced CNS disorders reviewed in ref. [22]. Collectively, the results of human and animal studies indicate that Stx-mediated host inflammatory and cellular stress responses may cause CNS damage, either directly (by acting on the intoxicated cells of the brain) or in response to cytokines produced in the CNS or in other primary Stx target organs, including the kidneys and intestines. 

The details of inflammatory and stress responses leading to Stxs-induced CNS injury are crucial for an understanding of toxin-mediated neuropathogenesis. Human brain endothelial cells (hBECs) are essential parts of the blood-brain barrier (BBB), which maintains CNS function by blocking the passage of macromolecules [192]. Primary hBECs have been shown to express both Gb3 and Gb4, and the toxin receptors partition into both detergent resistant and non-detergent resistant domains [193]. The capacity of toxin receptors to associate with detergent resistant membrane fractions, representing lipid rafts, is thought to be essential for toxin-mediated signaling [194]. Several in vitro studies have reported that exposure of hBECs to Stx1a caused pro-inflammatory responses by increasing the levels of cytokines that contribute to neuropathological symptoms [132,195]. Many of these cytokines are also associated with D+HUS pathogenesis.

The role of non-vascular CNS cell types in the neuropathogenesis of Stx-mediated disease is an area of intense scrutiny. Astrocytes are found in close proximity to hBECs and are responsible for maintaining the BBB [196]. Rat astrocytes produce TNF-α when stimulated with Stx1a + LPS [197]. Localized production of perivascular astrocyte-derived pro-inflammatory cytokines was shown to increase vascular permeability, increase hBEC susceptibility to toxin damage through elevated Gb3 expression, and increase adherence of neutrophils and platelets to endothelial cells. Treatment of Stx1a + LPS-stimulated astrocytes with the NF-κB inhibitor BAY 11-7082, or the TNF-α neutralizer Etanercept, ameliorated these pathological events, suggesting that TNF-α is a major determinant in neuropathogenesis. [197,198]. Presumably, then, various inflammatory mediators released by Stx-damaged hBECs, along with cytokines produced by newly recruited, activated leukocytes, exacerbate damage to the brain parenchyma as part of the disease progression leading ultimately to seizures, blindness or death. 

Rabbits administered Stx2a developed microvascular thrombosis with necrotic infarction and acute neuronal damage in the spinal cord gray matter. In contrast to the ischemic changes in the spinal cord, neuropathology in the brain was more subtle with evidence of scattered ischemic lesions and neuronal apoptosis. Neuronal damage, which correlated with the onset of microglial cell activation and elevated TNF-α and IL-1β mRNA transcripts in CNS parenchyma, progressed from the thalamus to pyramidal neurons in the hippocampus [199]. Whether neuronal damage is directly mediated by Stxs, or requires a localized pro-inflammatory response to manifest will require additional study. However, Pinto et al. [200] demonstrated that in a murine model following the administration of Stx2a, clinical signs and neurological alterations in the mouse striatum caused by a sub-lethal systemic dose of toxin can be prevented by treatment with the anti-inflammatory compound dexamethasone. Meuth et al. [201] used patch-clamp recording to show that Stx2a application resulted in strong depolarization of rat thalamic neurons. Addition of Stx2a to rat brain slices led to apoptosis of neuronal cells and astrocytes. The investigators also examined Gb3 synthase mRNA expression in many parts of the rat brain. Following toxin treatment, transcripts were found in all regions examined, although enhanced expression was found in the thalamus and brainstem. Interestingly, all of these changes manifested in female rats, but not male rats, suggesting gender specific differences in neuropathogenesis. Additional experiments will be necessary to characterize the mechanistic bases for gender differences. In a cohort study, Meuth et al. [201] performed cerebral magnetic resonance imaging (MRI) on 7 female patients from the *E. coli* O104:H4 outbreak in Germany. The patients had developed severe CNS complications including sustained epileptic seizures, stupor and coma. MRI showed symmetrical bilateral hyperintensities in the thalamus of all patients. Apparent diffusion coefficients were reduced suggesting restricted diffusion. The investigators interpreted the images as depicting cytotoxic edema with intact BBB, but impaired cellular metabolism. 

## 7. The Immunopathological Role of Stxs in Circulating Blood During HUS Pathogenesis 

As mentioned above, the common feature of histopathological changes in systemic disease caused by Stxs is damage to microvascular endothelial cells serving the kidneys and CNS. It is currently unclear why microvessels in these tissues should be selectively damaged, although Obrig et al. [202] reported that endothelial cells isolated from renal tissues express higher basal levels of Gb3. Te Loo et al. [203] showed that human neutrophils bind Stxs, but are not killed by the toxins. Based on these findings, the authors proposed a model in which the toxins “piggy back” on neutrophils. As neutrophils pass through tissues containing blood vessels with high Gb3 content, the toxins are transferred to endothelial cells. Later, the model was supported by clinical investigations demonstrating the presence of Stxs detectable on neutrophils in the circulating bloodstream of pediatric D+HUS patients [204] and experiments showing that Stx1a binds at 100-fold lower affinity to neutrophil receptors in comparison to cells expressing Gb3, and using flow cytometry to show that FITC-conjugated Stx1a is transferred from neutrophils to glomerular microvascular endothelial cells stimulated with TNF-α [205]. 

The recent advances in our understanding of mechanisms of toxin distribution in the bloodstream led to the search for the identity of the cell membrane carrier molecule(s) that transport Stxs from the intestinal submucosa to the kidneys and CNS during D+HUS progression in STEC-infected patients. The Sandvig laboratory used siRNA to knockdown expression of TLR4, originally characterized as a pattern recognition receptor for lipopolysaccharides, on the surface of colonic carcinoma cell lines and primary human umbilical vein endothelial cells [206]. Although these cells expressed Gb3, TLR4 depletion reduced toxin binding. Optimal toxin binding was restored when TLR4 was expressed on the cells. This same group showed that clathrin-dependent Stx1a holotoxin uptake by epithelial cell lines was greater than uptake of Stx1 B-subunits, suggesting that the presence of the A-subunit is crucial for optimal toxin internalization [207]. Collectively, these data suggest that TLR4 facilitates toxin binding, and the toxin A-subunit facilitates uptake by mechanisms that remain to be explored. In contrast to Gb3-expressing target cells, neutrophils do not express Gb3, yet they have been reported to be major toxin carrier cells. Furthermore, Stx interactions with neutrophils have been reported to involve A-subunit binding [208,209]. TLR4 has been reported to bind Stxs to the neutrophil surface without triggering toxin internalization and intracellular routing [138]. However, the leukocytes do not act as quiescent carrier cells. Instead, as Brigotti et al. [210] showed, brief incubation (90 min) of freshly isolated human monocytes or neutrophils with Stx1a resulted in release of the pro-inflammatory mediators TNFα, IL-1β, IL-6, G-CSF, CCL2, CCL4, and CXCL8 over the course of a 20 h. experimental period. Monocytes were the predominant producers of cytokines, even when values were normalized to reflect normal monocyte versus neutrophil cell numbers (5.0 × 10^5^ cells/mL vs. 3.5 × 10^6^ cells/mL, respectively) in the blood of healthy children. Brigotti et al. [210] also used monoclonal antibodies against Gb3 and TLR4 to investigate the roles of these receptors in inducing expression of CXCL8 in freshly isolated human peripheral blood monocytes and neutrophils. Antibodies directed against Gb3 inhibited Stx1a-induced chemokine expression, whereas anti-TLR4 antibody did not have a significant effect. Moreover, anti-TLR4 antibodies did not affect Stx1a-induced CXCL8 release, but did inhibit chemokine expression triggered by LPS. Thus, although TLR4 has been shown to play an essential role in the activation of innate immunity in response to bacterial lipopolysaccharides, signaling through Gb3 appears to be essential for cytokine and chemokine expression by human monocytes exposed to Stxs. 

A recent study used an LPS-primed Stx2a-challenged murine D+HUS model to investigate how the toxin is carried to the kidneys to cause HUS [211]. The experiments showed that circulating CD11b^+^ myeloid leukocytes isolated from LPS-primed Stx2a-intoxicated mice were more effective carriers of the toxin than cells isolated from unprimed mice. In response to stimulation by Stx2a and LPS, myeloid cells produced TNF-α and IL-1β, cytokines that stimulate inflammation, in part, by increasing tissue fluid flow from the bloodstream to the lymphatic circulation, thereby facilitating extravasation of phagocytes, complement proteins, and antibodies at the site of infection. By increasing fluid flow, these cytokines may enhance hematogenous spread of Stxs. TNF-α has been demonstrated to increase the levels of Stx receptors on endothelial cells by inducing galactosyl-transferase activity [199,212], suggesting that TNF-α induces one or more enzymes that are rate-limiting during synthesis of the glycolipid Stx-receptor. Finally, purified Stxs directly induced expression of TNF-α and IL-1β by murine and human monocytes in vitro, but the extent to which this phenomenon contributes to pathogenesis in vivo remains to be fully characterized. Numerous studies using animals treated with purified Stxs have reproduced the vascular lesions characteristic of HUS reviewed in ref. [22]. Clearly, in contrast to diarrheal disease which is multifactorial, Stxs are the principal virulence determinants responsible for initiating immunopathology, leading to life-threatening renal and CNS complications.

## 8. Conclusions and Future Perspectives

Attempts by our group and others to identify host cellular responses induced by Stxs have revealed a wide range of novel responses. If and to what extent these cellular responses are involved in pathogenesis by the toxins remain to be clarified. In particular, the characterization of the dynamic immune modulation and mediators of inflammation induced upon intoxication by Stxs will be necessary to define targets for intervention and derive coherent means to disrupt D+HUS disease progression. Clearly, the activation of innate immune responses by Stxs, including the stimulation of pro-inflammatory cytokine production, immune cell activation, and complement activation may cause primary tissue injury or exacerbate primary tissue injury caused by the toxins. Future studies should seek to identify potential targets for disruption of the innate immune response, thereby providing protection of target organs from damage induced by this family of potent, multifunctional toxins.
